# Acute Aortic Wall Stress Response to Handgrip Exercise in Aneurysmal versus Nondilated Ascending Thoracic Aortas

**DOI:** 10.1055/a-2875-3346

**Published:** 2026-05-25

**Authors:** Rachel J. Skow, Stephen J. Foulkes, Richard B. Thompson, Devyn Walesiak, Christopher Weinkauf, David Niederseer, Michael Sean McMurtry, Mark J. Haykowsky

**Affiliations:** 1 Integrated Cardiovascular Exercise Physiology and Rehabilitation (iCARE) Laboratory 3158 College of Health Sciences, University of AlbertaEdmonton, AlbertaCanada; 2 Heart Exercise and Research Trials Lab 85092 St Vincent's Institute of Medical ResearchMelbourneAustralia; 3 Baker Department of Cardiometabolic Health 3158 University of MelbourneParkville, VictoriaAustralia; 4 Department of Radiology and Diagnostic Imaging 3158 University of AlbertaEdmonton, AlbertaCanada; 5 89395 Hochgebirgsklinik, Medicine Campus DavosDavosSwitzerland; 6 Division of Cardiology, Faculty of Medicine and DentistryDepartment of Medicine 3158 College of Health Sciences, University of AlbertaEdmonton, AlbertaCanada

Physical activity guidelines for individuals with an ascending thoracic aortic aneurysm (ATAA) advise against performing heavy lifting or resistance exercise with a Valsalva maneuver (VM). 1 This is based on the concern that acute elevations in systolic blood pressure (SBP) and aortic wall stress (AWS) may increase the risk of acute aortic dissection; 2 however, no study has measured AWS during resistance exercise.

We measured AWS and its Laplace law determinants—SBP, ascending aorta diameter (AoD) and wall thickness (AWT) 3 4 —at rest and during submaximal dynamic handgrip (HG) exercise in eight individuals with an ATAA (69 ± 11 years, 3 females) and 11 age-matched controls (68 ± 6 years, 4 females). This study was approved by the University of Alberta Research Ethics Board (Pro00139002). Participants with ATAA were included if their AoD was >40 mm and were clinically stable with no indications for aortic surgery. Control participants had no known history of aortic enlargement, and both groups had a resting blood pressure below 140/90 mm Hg.

**Fig. 1:**
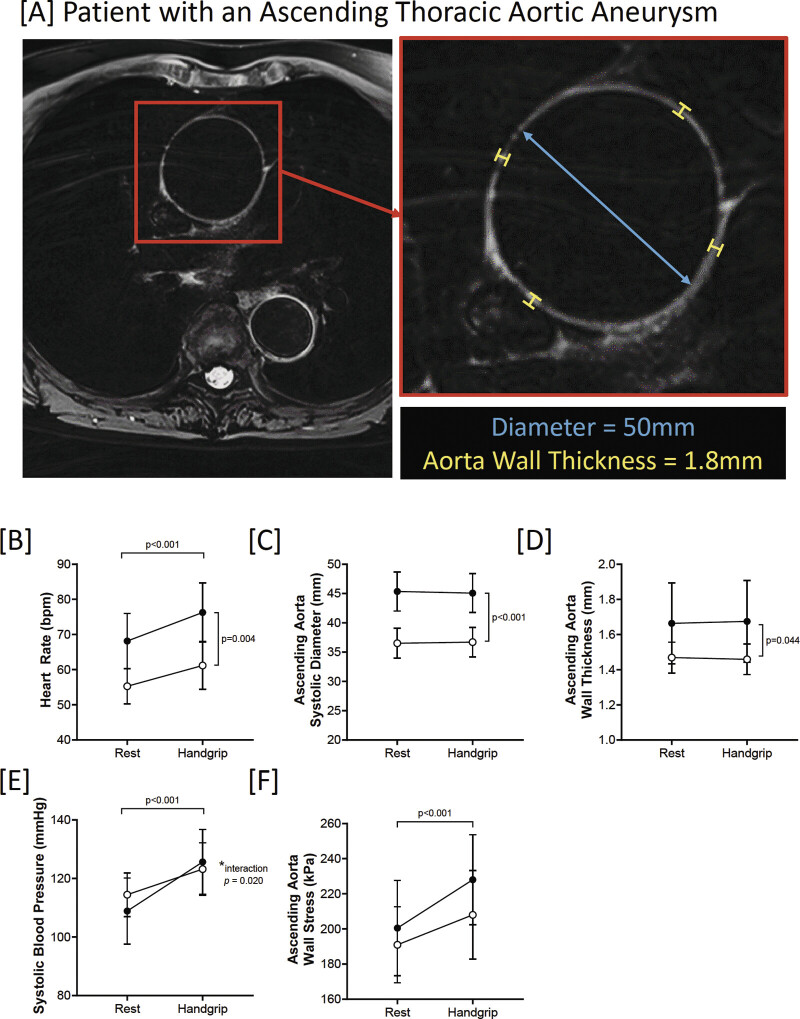
Ascending aorta and hemodynamic responses to handgrip exercise in patients with an ascending thoracic aortic aneurysm (ATAA; black symbols) compared with age-matched controls (white symbols). Data are presented as mean and 95% confidence interval (error bars). Effects of group (ATAA vs. Control), condition (Rest vs. Handgrip), and the group by condition interaction were compared by two-way repeated measures ANOVA using GraphPad Prism (v10.6.1). Significance was set at *p* < 0.05. Magnetic resonance imaging measurement of ascending thoracic aorta diameter and wall thickness ( **A** ). There were significant main effects of group for heart rate ( **B** ) ascending aorta diameter and wall thickness ( **C** and **D** ), and main effects of condition for heart rate ( **B** ), systolic blood pressure ( **E** ), and ascending aorta wall stress ( **F** ). *There was an interaction effect for systolic blood pressure such that patients with ATAA had larger increases in this outcome during handgrip exercise ( **E** ; *p* = 0.020). ANOVA, analysis of variance.

Three maximal HG contractions (dominant hand) were averaged to establish the submaximal exercise intensity (∼40% of maximum). Baseline measures were obtained in a 3T MRI scanner (Siemens Prisma, Siemens Engineering), with heart rate (HR, single-lead electrocardiography) and brachial (cuff) blood pressure monitoring. Imaging of the ascending aorta was performed as previously described 4 ( Fig. 1A ). Submaximal HG exercise consisted of 2-second contraction/relaxation cycles for 5 minutes performed without a VM. Exercise AoD was measured at the fourth minute of exercise, and exercise AWT was calculated from resting AWT and measured changes in AoD between rest and exercise as previously described. 4 AWS was calculated as: (0.133 × SBP × AoD)/(2 × AWT). 4 Comparisons were performed using two-way repeated-measures analysis of variance with α set at *p* < 0.05.

No group differences were found for HG exercise intensity (ATAA: 46 ± 5% vs. Control: 44 ± 5%, *p* = 0.491). Significant main effects of “group” were found for HR, AoD, and AWT, all of which were higher in ATAA than controls ( Fig. 1B–D ). Significant main effects of “condition” were found for HR, SBP, and AWS, with values during HG exercise being greater than rest ( Fig. 1B, E–F ). An interaction effect was found for SBP such that the increase was larger in the ATAA group ( *p* = 0.020).

The major finding is that AWS during submaximal dynamic HG exercise in ATAA was not significantly different than controls and was 4.4-fold lower than the maximal tensile strength (rupture threshold) measured in ex vivo aneurysmal ascending aortic tissue (equal to 1,000 Kpa). 5 Contextualizing the observed HG exercise AWS values against published aortic maximal tensile strength values provides reassurance that moderate-intensity dynamic HG exercise is well below catastrophic levels of AWS in our cohort. 5

A limitation of our study was that blood pressure was measured noninvasively during HG exercise. Prior work has demonstrated that noninvasive blood pressure measures underestimate invasive measures by 12 to 14% during resistance exercise; 6 therefore, it is possible that our measured SBP and AWS may have been higher than our reported values for each group. However, this limitation would not alter our primary finding of no difference in AWS between groups at rest or during HG exercise

In summary, the compensatory role of increased AWT in individuals with an ATAA provides a protective buffer against transient rises in SBP during dynamic HG exercise performed without a VM. Future studies should examine AWS responses during dynamic resistance exercise involving larger muscle groups and across a wide spectrum of exercise intensities to determine the thresholds at which AWS increases may begin to pose clinical concern.
